# Evolutionary conservation of motifs within *vanA* and *vanB* of vancomycin-resistant enterococci

**DOI:** 10.14202/vetworld.2022.2407-2413

**Published:** 2022-10-13

**Authors:** Aylin Memili, Naseer Kutchy, Olubumi A. Braimah, Olanrewaju B. Morenikeji

**Affiliations:** 1 Department of Genetics, University of North Carolina at Chapel Hill, Chapel Hill, North Carolina, United States; 2 Department of Anatomy, Physiology, Pharmacology, School of Veterinary Medicine, St. George’s University, Grenada, West Indies; 3 Division of Biological Health Sciences, University of Pittsburgh, Bradford, Pennsylvania, United States

**Keywords:** antibiotic resistance, bioinformatics, *Enterococcus*, evolution, public health

## Abstract

**Background and Aim:**

Global Health is threatened by the rapid emergence of multidrug-resistant bacteria. Antibiotic resistomes rapidly evolve, yet conserved motifs elucidated in our study have the potential for future drug targets for precision medicine. This study aimed to identify conserved genetic sequences and their evolutionary pathways among vancomycin-resistant *Enterococcus* species such as *Enterococcus faecium* and *Enterococcus faecalis*.

**Materials and Methods:**

We retrieved a total of 26 complete amino acid and nucleotide sequences of resistance determinant genes against vancomycin (*vanA* and *vanB)*, streptomycin (aac-aah), and penicillin (*pbp5*) from the publicly available genetic sequence database, GenBank. The sequences were comprised of bacteria classified under the genera of *Enterococcus*, *Staphylococcus*, *Amycolatopsis*, *Ruminococcus*, and *Clostridium*. Sequences were aligned with Clustal Omega Multiple Sequence Alignment program and Percent Identity Matrices were derived. Phylogenetic analyses to elucidate evolutionary relationships between sequences were conducted with the neighbor-end joining method through the Molecular Evolutionary Genetics Analysis (MEGAX) software, developed by the Institute of Molecular Evolutionary Genetics at Pennsylvania State University. Subsequent network analyses of the resistance gene, *vanB*, within *E. faecium* were derived from ScanProsite and InterPro.

**Results:**

We observed the highest nucleotide sequence similarity of *vanA* regions within strains of *E. faecium* (100%) and *E. faecalis* (100%). Between *Enterococcus* genera, we continued to observe high sequence conservation for *vanA* and *vanB*, up to 99.9% similarity. Phylogenetic tree analyses suggest rapid acquisition of these determinants between strains within *vanA* and *vanB*, particularly between strains of *Enterococcus* genera, which may be indicative of horizontal gene transfer. Within *E. faecium*, Adenosine 5’-Triphosphate (ATP)-Grasp and D-ala-D-ala ligase (Ddl) were found as conserved domains of *vanA* and *vanB*. We additionally found that there is notable sequence conservation, up to 66.67%, between resistomes against vancomycin and streptomycin among *E. faecium*.

**Conclusion:**

Resistance genes against vancomycin have highly conserved sequences between strains of *Enterococcus* bacteria. These conserved sequences within *vanA* and *vanB* encode for ATP-Grasp and Ddl motifs, which have functional properties for maintaining cell wall integrity. High sequence conservation is also observed among resistance genes against penicillin and streptomycin, which can inform future drug targets for broader spectrum therapies.

## Introduction

Antibiotic resistance (AR) continues to threaten global public health, and the crisis is exacerbated by the rapid emergence of multidrug-resistant (MDR) bacteria. Over a million global cases of MDR bacteria were reported in the year 2020 [1], and MDR bacteria are rapidly being transmitted across habitats of humans, animals, and the environment [2–3]. Several recent investigations reported the emergence of multidrug-resistant bacterial pathogens from different origins that increase the necessity for the proper use of antibiotics [4]. In addition, improving the routine application of antimicrobial susceptibility testing to detect the antibiotic of choice as well as the screening of the emerging MDR strains is imperative for tracking resistance growth.

In addition to improving the detection of MDR strains, there is an increasing focus on understanding the resistomes or resistance networks of MDR bacteria. The concept of resistomes stems from the emergence of diverse AR genes (ARGs) from bacterial gene mutations induced by antibiotic exposure and interconnected by functional pathways [5]. Several Gram-positive and Gram-negative bacteria have already developed resistance to many lifesaving antibiotics [6]. This situation is worsened due to the ineffectiveness of antibiotics in the treatment of infections caused mainly by bacteria carrying ARG. Because of the wide and excessive usage of antibiotics, it is possible that ARGs circulate among animal, human, and environmental microbes, thus presenting a universal threat to Global Health [7–9].

There are several ways antibiotics have been misused, which contribute to the circulation of MDR across different habitats. For example, there have been numerous reports of over- or misuse of antibiotics in livestock production, thereby exacerbating the problems of AR [10]. *Enterococcus faecium* strains have been isolated and identified in animals, humans, and environment, incorporating several multidrug resistance mechanisms as seen with *E. faecium* and *Enterococcus faecalis* [11] which are prevalent among livestock species [12].

On exposure to antibiotics, bacteria undergo mutations and develop remarkable multidrug resistance mechanisms such as cleaving the antibiotic, modifying the target site(s) in bacteria, or pumping the antibiotics out of the cell [13]. Studies on vancomycin-resistant *Staphylococcus aureus* (VRSA) remain limited to case study observations. However, efforts to genotype these strains have revealed that specific sequence regions are upregulated and potentially contribute to their resistance. Remarkably, the strains of VRSA found in human infections have also been detected in livestock as well as other animal reservoirs with high transmissibility to humans [14, 15].

Conserved motifs within these resistance strains include a combination of horizontally transmitted genes that activate the transcription and translation of protein products to overcome AR [13, 16]. Several AR mechanisms among enterococci have been elucidated. Modifications to the cell wall are common ways enterococci evade precious antibiotics. For example, resistance against vancomycin is conferred through several virulence factors, such as glycopeptide resistance operons, which disrupt the D-ala-D-ala and D-ala-D-lac cross-links in the peptidoglycan cell wall [17]. Rapid acquisition of AR between *E. faecalis* and *E. faecium* can be credited to the horizontal gene transfer of these resistance genes, which is facilitated by transposons [18]. The implications of rapid resistance gene transfer between enterococci are the dwindling treatments available to combat infections such as gastrointestinal tract infections, meningitis, and bacteremia [19].

We focused on evolutionary pathways of vancomycin resistance among *Enterococcus*, as the transmission of species within this group is known to travel rapidly through mobile transposons and plasmids. Here, we tested whether conserved regions within ARG can elucidate markers of evolutionary transmission of motifs involved in vancomycin resistance. By assessing the commonalities between resistant bacteria, we aimed to provide better insights toward finding drug targets that can combat the public health crisis of AR.

## Materials and Methods

### Ethical approval

The study did not involve direct human subjects and was solely conducted on publicly available data. For these reasons, ethics approval does not apply to the following study.

### Study period and location

This study was conducted from January 2016 to December 2020. All computational analyses were performed on personal computing devices of authors at the University of North Carolina in Chapel Hill, Chapel Hill, North Carolina, United States, at St. George’s University, Grenada, West Indies, and at the University of Pittsburgh, Bradford, Pennsylvania, United States.

### Sample derivation

We downloaded 26 ARG’ amino acid and DNA sequences against vancomycin (*vanA* and *vanB)*, streptomycin (aac-aah), and penicillin (*pbp5*) from the publicly available genetic sequence database, GenBank [20]. The sequences were among genera of *Enterococcus*, *Staphylococcus*, and *Amycolatopsis*, *Ruminococcus*, and *Clostridium*.

### Analysis of sequence alignment

Clustal Omega [21] was used to perform multiple sequence alignment with seeded guide trees to determine the Percent Identity Matrix (PIM) among the sequences. Likewise, gene sequences of *vanA*, *vanB*, *pbp5*, and *aac-aah* of *Enterococcus* were aligned and compared to ascertain PIM and conserved resistomes among the strains.

### Phylogenetic analysis of protein sequences

The sequences were imported to MEGAX software (https://www.megasoftware.net/) for subsequent analyses. Phylogenetic analysis was performed with neighbor-joining clustering method [22] and bootstrap with 500 iterations on MEGA software [23]. Protein sequences from GenBank were inserted into the phylogenetic analysis option in MEGAX, and the neighbor-joining method was selected to measure the evolutionary distance between strains.

### Network analysis of vancomycin resistance genes

To determine important sites that may be relevant through evolutionary conservation, we scanned for conserved motifs among the amino acid sequences of *vanA* and *vanB* in *E. faecium* with the combined use of ScanProsite [24, 25] and InterPro, an online program that helps analyze protein sequences and classification [26]. In addition, interactome analyses were performed using Search Tool for the Retrieval of Interacting Genes/Proteins (STRING v11.5) (https://string-db.org/), which is manufactured by ELIXIR Core Data Resources [27]. STRING was applied to depict the functional properties and protein-protein interactions of *vanA* and *vanB* and other proteins.

## Results

### Sequence conservation between strains

We observed notable conservation across the nucleotide sequences of *vanA* resistance genes among bacterial species, including *E. faecalis* and *E. faecium* (Table-1). The sequence similarity is particularly high within *E. faecalis* and *E. faecium* strains (100%) and remains high between these strains (99.9%). The sequence similarity of *vanA* decreased between enterococci and bacteria found in environmental isolates, such as *Amycolatopsis*.

**Table-1:** The percent identity matrix of *vanA* nucleotides across vancomycin-resistant genera.

Genera	1	2	3	4	5	6	7	8	9	10	11	12
1. *E. faecalis*	100.0	41.9	46.7	44.6	45.2	45.4	45.6	45.2	46.2	45.0	45.0	45.0
2. *Amycolatopsis* spp. WAC1375	41.9	100.0	50.0	57.2	56.5	57.8	55.0	57.8	58.3	57.4	57.4	57.4
3. *E. faecium* Efm/Chennai. IND/090	46.7	50.0	100.0	95.5	95.5	95.5	99.0	95.5	95.5	95.5	95.5	95.5
4. *E. faecium* plasmid transposon Tn1546	44.6	57.2	95.5	100.0	99.9	99.9	99.8	99.9	99.9	99.9	99.9	99.9
5. *E. faecium* F135/41	45.2	56.5	95.5	99.9	100.0	100.0	100.0	100.0	100.0	100.0	100.0	100.0
6. S. haemolyticus	45.4	57.8	95.5	99.9	100.0	100.0	100.0	100.0	100.0	100.0	100.0	100.0
7. *E. faecium* VREF-P2	45.6	55.0	99.0	99.8	100.0	100.0	100.0	100.0	100.0	100.0	100.0	100.0
8. S. aureus ST1RCGLD-IPI	45.2	57.8	95.5	99.9	100.0	100.0	100.0	100.0	100.0	100.0	100.0	100.0
9. *E. faecalis* plasmid pWZ909	45.0	57.4	95.5	99.9	100.0	100.0	100.0	100.0	100.0	100.0	100.0	100.0
10. *E. faecalis* plasmid pWZ1668	45.0	57.4	95.5	99.9	100.0	100.0	100.0	100.0	100.0	100.0	100.0	100.0
11. *E. faecium* ZP2298	45.0	57.4	95.5	99.9	100.0	100.0	100.0	100.0	100.0	100.0	100.0	100.0
12. *E. faecalis* plasmid pSL1	45.0	57.4	95.5	99.9	100.0	100.0	100.0	100.0	100.0	100.0	100.0	100.0

The table presents a matrix of representative genera of interest, and the number of the rows corresponds to the number of the columns. Numeric scores are assigned to the aligned sequences, accounting for variation in length of sequence. Similarity scores are assigned according to the structural similarity of sequence. *E. faecalis*=*Enterococcus faecalis*, *E. faecium=Enterococcus faecium*

**Figure-1:**
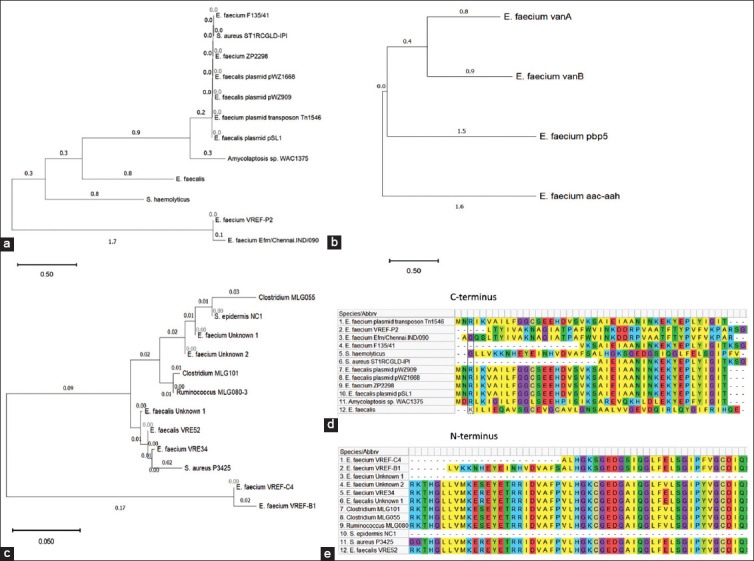
Analysis of evolutionary conservation across bacterial species and strains using phylogenetic analysis. (a) Evolutionary pattern showing the development of antibiotic resistance in different bacterial species and strains for *vanA* as an antibiotic determinant in them. (b) Evolutionary pattern showing the development of antibiotic resistance in different bacterial species and strains for *vanB* as an antibiotic determinant in them, (c) The phylogenic tree consists of *Enterococcus faecium* strains with resistance determinants against vancomycin, streptomycin, and penicillin. The phylogenetic trees were developed using neighbor-joining method. The optimal tree with the sum of branch length = 2.01420299 is shown. The percentage of replicate trees in which the associated taxa clustered together in the bootstrap test (500 replicates) is shown above the branches. (d) Amino acid sequence alignment of *vanA* across different bacterial species harboring *vanA* antibiotic resistance determinant. (e) Amino acid sequence alignment of *vanB* across different bacterial species harboring *vanB* antibiotic resistance determinant. Similar shading of the amino acids indicates similarities in solubility properties.

The amino acid sequences within species for both *vanA* and *vanB* are highly similar (Figure-1d and e), as indicated by the similar shading. Although, similarities in sequence alignment were higher within the C-terminus of *vanA* and N-terminus of *vanB* genes. When the resistance genes against vancomycin, penicillin, and streptomycin were compared within *E*. *faecium*, the PIM yielded the lowest sequence similarity between *vanA* and *pbp5 (*46.58%) and the highest sequence similarity between *vanA* and *vanB* (100%), as shown in Table-2.

**Table-2:** Percent identity matrix of four antibiotic resistance determinants of *Enterococcus faecium*.

Determinants	*vanA*	*vanB*	*pbp5*	*aac-aahF*
*vanA*	100.00	100.00	46.58	66.67
*vanB*	100.00	100.00	46.90	66.67
*pbp5*	46.58	46.90	100.00	47.60
*aac-aah*	66.67	66.67	47.60	100

Numeric scores are assigned to the aligned sequences, accounting for variation in length of the sequence. Similarity scores are assigned according to the structural similarity of the sequence

### Phylogenetic analysis of evolutionary distance

Evolutionary analyses of *vanA* amino acid sequences across the bacterial species show that *E. faecalis* and *E. faecium* developed resistance within similar time periods (Figure-1a), as indicated by the shortest distances between branches on the phylogenetic tree between the enterococci. Similar observations were made for *vanB*, indicating that *E. faecalis* and *E. faecium* quickly developed resistance later in the evolution tree, as indicated by the short distance between new strains and their former evolutionary counterparts (Figure-1b). The enterococci can be found within the origins of resistance genes against vancomycin as well as at the most recent resistance strain, as indicated by its location at the bottom and top of the phylogenetic trees. Interestingly, as seen in our phylogenetic tree analyses, *E. faecium* developed vancomycin resistance first through *vanB and* then through *vanA* genes (Figure-1c), as indicated by the position on the tree. The findings suggest that resistance against streptomycin originated before the resistance against vancomycin (Figure-1c).

### Functional properties of conserved regions

**Figure-2:**
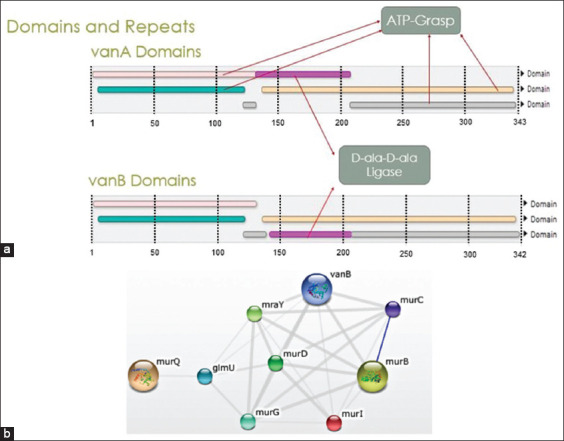
(a) InterPro display of entry matches to protein sequences for *vanA* and *vanB*. Different domains are assigned a unique color. Same color for D-ala-D-ala ligase sequence identifies the conserved region. (b) The interactome of *vanB* identifies proteins that are involved in similar pathways of *vanB*. Connections, drawn by gray lines, indicate the existence of a connection. The blue line represents the strongest connection, which is between two Mur proteins.

We demonstrate that both *vanA* and *vanB* genes translate to important conserved domains: ATP-Grasp and D-ala-D-ala ligase (Ddl) (Figure-2a). However, orientation of *vanA* and *vanB* Ddl domains differs. Our interactome analysis of *vanA* and *vanB* in *E. faecium* indicates that only the functional properties of *vanB* are concentrated within the bacterial cell wall formation (Figure-2b). The main interactions predicted by the estimated model show the involvement of *Mur* enzymes with *vanB* functionality.

## Discussion

Uncovering the evolutionarily conserved resistance mechanisms among microbes are critical for understanding the transmission of ARG as well as the effects on Global Health. We identify regions of amino acid sequence conservation within vancomycin-resistant genes, thus underpinning the patterns of selection pressures associated with their transmission, emergence, and evolution. While *Enterococcus* strains resistant to vancomycin are commonly found in the clinical setting, strains within *Amylocolaptosi*s genera are commonly found within soil environments [28], which can possibly explain the low similarity between the *vanA* gene sequences of this strain versus *Enterococcus*. Sequence similarity between resistance determinants across vancomycin, streptomycin, and penicillin was also observed between nucleotide sequences, which provides insight into the conserved regions that could be targeted to achieve a broader spectrum of antibiotic efficacy.

Evolutionary analysis of *vanA* and *vanB* indicates a swift development of resistance between *Enterococcus* strains against vancomycin. This observation suggests a quick shuffling/flow of ARG among bacteria which could be used to monitor transmission and emergence in the Global Health program. Gorrie *et al*. [29] reported the increased prevalence of MDR *E. faecium* strains against topline antibiotics, including penicillin. This is in line with our observation based on the percent similarities between resistance determinants of *E. faecium*. We find a gradual acquisition of resistance against antibiotics within *E. faecium* strains, suggesting that the earliest resistant determinant against streptomycin appears to be evolutionarily distant as compared to the other resistome determinants. The split between resistome determinants against penicillin and vancomycin suggests that more resistance mechanisms against vancomycin developed after penicillin resistance among enterococci. These results also suggest that these resistance determinants against vancomycin and penicillin evolved in a closer timeline compared to that of streptomycin, as indicated by the shorter distance between the branches of *vanA*, *vanB*, and *pbp5*, when compared to *aac-aph*.

These results are significant because they shed light on the functional characteristics of these conserved motifs and demonstrate the conservation of two important domains: ATP-Grasp and Ddl, among *vanA* and *vanB* genes. Differences in the orientation of these domains might be indicative of the strength and diversity of resistance between *vanA* and *vanB*. The functionality of Ddl ligase depends on its ability to acquire ATP to catalyze reactions for maintaining the integrity of the bacterial cell wall, which makes it within the ATP-grasp enzyme superfamily [30]. While Ddl regions have previously been shown to be targeted by antibiotics [31], the diversity of the roles that ATP-grasp enzymes play in several metabolic pathways leaves several targets yet to be discovered.

Our interactome analyses also confirm the role of *vanB* involvement with the development of cell wall structure per interactions with *Mur* enzymes known to be involved in catalyzing the reactions for peptidoglycan construction by transferring peptidyl residues to form Uridine diphosphate (UDP)-N-acetylmuramyl pentapeptide [32]. It is worth noting that *in vitro* studies have targeted these Mur regions for inactivation within antimicrobials and possibly led to the discovery of effective new compounds [33–35]. The association of *vanB* with potential drug targets is intriguing for the development of potent tools for precision medicine.

The targets we have identified align with the known virulence factors of enterococci, which include mechanisms of cell wall modifications, ribosomal modifications, and cleavage of antibiotic-binding sites [17]. As the frequency of MDR enterococci is increasing among isolates from humans and animals [36], it is imperative that mobile transposons and operons, agents which facilitate rapid horizontal gene transfer, are identified by considering their evolutionary patterns. Subsequent analyses on the functional pathways of Ddl and ATP-Grasp are needed to understand how these elements are transferred between enterococci and whether these common domains have similar modes of transportation.

## Conclusion

This study identified that resistance genes against vancomycin are rapidly acquired within *Enterococcus* strains and have high sequence similarity between these strains. The conserved regions indicate the persistence of motifs, ATP Grasp, and Ddl, consistently found within *vanA* and *vanB*. These motifs may serve as drug targets once their modes of transportation and subsequent functional pathway analyses are conducted to understand how they contribute to resistance.

## Data Availability

The sequences used for this study can be available from the corresponding author on a reasonable request.

## Authors’ Contributions

AM, NK, and OBM: Conceptualized the research project, designed the project, and developed the methodology for the study. AM, NK, and OBM: Implemented the project and oversaw the writing of the original draft. AM, NK, OAB, and OBM: Reviewed and edited the manuscript. All authors have read and approved the final manuscript.

## References

[1] World Health Organization (2020). Antibiotic Resistance.

[2] Algammal A.M, Hashem H.R, Al-Otaibi A.S, Alfifi K.J, El-Dawody E.M, Mahrous E, Hetta H.F, El-Kholy A.W, Ramadan H, El-Tarabili R.M (2021). Emerging MDR-*Mycobacterium avium* subsp. Avium in house-reared domestic birds as the first report in Egypt. BMC Microbiol.

[3] Algammal A.M, Wahdan A, Elhaig M.M (2019). Potential efficiency of conventional and advanced approaches used to detect *Mycobacterium bovis* in cattle. Microb. Pathog.

[4] Ali Abushaheen M, ?Muzaheed M, Fatani A.J, Alosaimi M, Mansy W, George M, Acharya S, Rathod S, Divakar D.D, Jhugroo C, Vellappally S, Khan A.A, Shaik J, Jhugroo P (2020). Antimicrobial resistance, mechanisms and its clinical significance. Dis. Mon.

[5] Kim D.W, Cha C.J (2021). Antibiotic resistome from the one-health perspective:Understanding and controlling antimicrobial resistance transmission. Exp. Mol. Med.

[6] Acharya Y, Bhattacharyya S, Dhanda G, Haldar J (2022). Emerging roles of glycopeptide antibiotics:Moving beyond gram-positive bacteria. ACS Infect. Dis.

[7] Zhuang M, Achmon Y, Cao Y, Liang X, Chen L, Wang H, Siame B.A, Leung K.Y (2021). Distribution of antibiotic resistance genes in the environment. Environ. Pollut.

[8] Gwenzi W, Shamsizadeh Z, Gholipour S, Nikaeen M (2022). The air-borne antibiotic resistome:Occurrence, health risks, and future directions. Sci. Total Environ.

[9] Chen C, Pankow C.A, Oh M, Heath L.S, Zhang L, Du P, Xia K, Pruden A (2019). Effect of antibiotic use and composting on antibiotic resistance gene abundance and resistome risks of soils receiving manure-derived amendments. Environ. Int.

[10] Guo K, Zhao Y, Cui L, Cao Z, Zhang F, Wang X, Feng J, Dai M (2021). The influencing factors of bacterial resistance related to livestock farm:Sources and mechanisms. Front. Anim. Sci.

[11] Thu W.P, Sinwat N, Bitrus A.A, Angkittitrakul S, Prathan R, Chuanchuen R (2019). Prevalence, antimicrobial resistance, virulence gene, and class 1 integrons of *Enterococcus faecium* and *Enterococcus faecalis* from pigs, pork and humans in Thai-Laos border provinces. J. Global Antimicrob. Resist.

[12] De Jong A, Simjee S, Garch F.E, Moyaert H, Rose M, Youala M, Dry M (2018). Antimicrobial susceptibility of enterococci recovered from healthy cattle, pigs and chickens in nine EU countries (Eassa study) to critically important antibiotics. Vet. Microbiol.

[13] Peterson E, Kaur P (2018). Antibiotic resistance mechanisms in bacteria:Relationships between resistance determinants of antibiotic producers, environmental bacteria, and clinical pathogens. Front. Microbiol.

[14] Bhattacharyya D, Banerjee J, Bandyopadhyay S, Mondal B, Nanda P.K, Samanta I, Mahanti A, Das A.K, Das G, Dandapat P, Bandyopadhyay S, Mondal B, Nanda P.K, Samanta I, Mahanti A, Das A.K, Das G, Dandapat P, Bandyopadhyay S (2016). First report on vancomycin-resistant *Staphylococcus aureus* in bovine and caprine milk. Microbial. Drug Resist.

[15] Moreno-Grúa E, Pérez-Fuentes S, Muñoz-Silvestre A, Viana D, Fernández-Ros A.B, Sanz-Tejero C, Corpa J.M, Selva L (2018). Characterization of livestock-associated 0methicillin-resistant *Staphylococcus aureus* isolates obtained from commercial rabbitries located in the Iberian peninsula. Front. Microbiol.

[16] Ellabaan M, Munck C, Porse A, Imamovic L, Sommer M.O.A (2021). Forecasting the dissemination of antibiotic resistance genes across bacterial genomes. Nat. Commun.

[17] García-Solache M, Rice L.B (2019). The *Enterococcus*:A model of adaptability to its environment. Clin. Microbiol. Rev.

[18] Bligh S.W, Drake A.F, Sadler P.J (1990). Nuclear magnetic resonance and circular dichroism spectroscopic studies of copper complexation in blood plasma. Biochem. Soc. Trans.

[19] Said M.S, Tirthani E, Lesho E (2022). Enterococcus Infections.

[20] National Center for Biotechnology Information (1988). National Library of Medicine (US).

[21] Sievers F, Wilm A, Dineen D, Gibson T.J, Karplus K, Li W, Lopez R, McWilliam H, Remmert M, Söding J, Thompson J.D, Higgins D.G (2011). Fast, scalable generation of high-quality protein multiple sequence alignments using Clustal Omega. Mol. Syst. Bio.

[22] Tamura K, Nei M, Kumar S (2004). Prospects for inferring very large phylogenies by using the neighbor-joining method. Proc. Natl. Acad. Sci. U. S. A.

[23] Kumar S, Stecher G, Li M, Knyaz C, Tamura K (2018). MEGA X:Molecular evolutionary genetics analysis across computing platforms. Mol. Biol. Evol.

[24] De Castro E, Sigrist C.J.A, Gattiker A, Bulliard V, Langendijk-Genevaux P.S, Gasteiger E, Bairoch A, Hulo N (2006). ScanProsite:Detection of PROSITE signature matches and ProRule-associated functional and structural residues in proteins. Nucleic Acids Res.

[25] Sigrist C.J.A, de Castro E, Cerutti L, Cuche B.A, Hulo N, Bridge A, Bougueleret L, Xenarios I (2012). New and continuing developments at PROSITE. Nucleic Acids Res.

[26] Jones P, Binns D, Chang H.Y, Fraser M, Li W, McAnulla C, McWilliam H, Maslen J, Mitchell A, Nuka G, Pesseat S, Quinn A.F, Sangrador-Vegas A, Scheremetjew M, Yong S.Y, Lopez R, Hunter S (2014). InterProScan 5:Genome-scale protein function classification. Bioinformatics.

[27] Szklarczyk D, Gable A.L, Lyon D, Junge A, Wyder S, Huerta-Cepas J, Simonovic M, Doncheva N.T, Morris J.H, Bork P, Jensen L.J, von Mering C (2019). STRING v11:Protein-protein association networks with increased coverage, supporting functional discovery in genome-wide experimental datasets. Nucleic Acids Res.

[28] Tan G.Y, Ward A.C, Goodfellow M (2006). Exploration of *Amycolatopsis* diversity in soil using genus-specific primers and novel selective media. Syst. Appl. Microbiol.

[29] Gorrie C, Higgs C, Carter G, Stinear T.P, Howden B (2019). Genomics of vancomycin-resistant *Enterococcus faecium*. Microb. Genom.

[30] Pederick J.L, Thompson A.P, Bell S.G, Bruning J.B (2020). D-Alanine-d-alanine ligase as a model for the activation of ATP-grasp enzymes by monovalent cations. J. Biol. Chem.

[31] Ameryckx A, Pochet L, Wang G, Yildiz E, Saadi B.E, Wouters J, Van Bambeke F, Frédérick R (2020). Pharmacomodulations of the benzoyl-thiosemicarbazide scaffold reveal antimicrobial agents targeting d-alanyl-d-alanine ligase in bacteria. Eur. J. Med. Chem.

[32] El Zoeiby A, Sanschagrin F, Levesque R.C (2003). Structure and function of the Mur enzymes:Development of novel inhibitors. Mol. Microbiol.

[33] Zaveri K, Kiranmayi P (2017). Screening of potential lead molecule as novel mure inhibitor:Virtual screening, molecular dynamics and *in vitro* studies. Curr. Comput. Aided Drug Des.

[34] Kouidmi I, Levesque R.C, Paradis-Bleau C (2014). The biology of Mur ligases as an antibacterial target. Mol. Microbiol.

[35] Hrast M, Turk S, Sosič I, Knez D, Randall C.P, Barreteau H, Contreras-Martel C, Dessen A, O'Neill A.J, Mengin-Lecreulx D, Blanot D, Gobec S (2013). Structure-activity relationships of new cyanothiophene inhibitors of the essential peptidoglycan biosynthesis enzyme MurF. Eur. J. Med. Chem.

[36] De Araujo G.O, Huff R, Favarini M.O, Mann M.B, Peters F.B, Frazzon J, Frazzon A.P.G (2020). Multidrug resistance in enterococci isolated from wild pampas foxes (*Lycalopex gymnocercus*) and geoffroy's cats (*Leopardus geoffroyi*) in the Brazilian pampa biome. Front. Vet. Sci.

